# Which features of advanced head and neck basal cell carcinoma are associated with perineural invasion?^☆^

**DOI:** 10.1016/j.bjorl.2016.01.009

**Published:** 2016-04-19

**Authors:** André Bandiera de Oliveira Santos, Natália Martins Magacho de Andrade, Lenine Garcia Brandão, Claudio Roberto Cernea

**Affiliations:** Universidade de São Paulo (USP), São Paulo, SP, Brazil

**Keywords:** Perineural invasion, Basal cell carcinoma, Head and neck, Carcinoma basocelular, Invasão perineural, Cabeça e pescoço

## Abstract

**Introduction:**

Perineural invasion is a unique route for tumor dissemination. In basal cell carcinomas, the incidence is low, but increases in advanced cases. Its importance is recognized but not fully understood.

**Objective:**

To compare head and neck basal cell carcinomas with and without perineural invasion.

**Methods:**

A retrospective medical chart review of multidisciplinary surgeries for basal cell carcinomas that required a head and neck surgery specialist in a tertiary referral center was performed. Clinical-demographics and histopathological features were analyzed.

**Results:**

Of 354 cases, perineural invasion was present in 23.1%. Larger tumors and morpheaform subtype were statistically related to perineural invasion. Nodular and superficial subtypes were less frequent in positive cases. No significant difference was found in gender, age, ulceration, location, and mixed histology.

**Conclusion:**

In this series of selected patients with basal cell carcinomas submitted to major resections, perineural invasion was clearly related to morpheaform subtype and to larger tumors. Other classically associated features, such as location in high-risk mask zone of the face, male gender and mixed histology, were not so strongly linked to perineural invasion.

## Resumo

**Introdução:**

A invasão perineural é uma via independente de disseminação tumoral. Em carcinomas basocelulares, a incidência é baixa, mas aumenta em casos avançados. Sua importância é reconhecida mas não completamente compreendida.

**Objetivo:**

Comparar os carcinomas basocelulares com e sem invasão perineural.

**Método:**

Estudo retrospectivo de revisão de prontuários de pacientes submetidos a cirurgias de grande porte, conduzida por equipes multidisciplinares em centro terciário de referência de câncer. Características demográficas e histopatológicas foram analisadas.

**Resultados:**

De 354 casos; 23,1% apresentaram invasão perineural. Tumores com dimensão maior e subtipo esclerodermiforme foram significativamente mais associados a invasão perineural. Os subtipos nodular e superficial foram menos frequentes em casos positivos. Não houve diferença relacionada a sexo, idade, ulceração, localização e histologia mista.

**Conclusão:**

Nessa série de casos selecionados de carcinomas basocelulares submetidos a grandes ressecções, a invasão perineural foi claramente relacionada ao subtipo esclerodermiforme e tumores de maior dimensão. Outros fatores classicamente associados, como localização em zona de alto risco, sexo masculino e histologia mista não apresentaram essa associação de modo significativo.

## Introduction

Perineural invasion was recently recognized as a unique route of tumor spread, regardless of the lymphatic and hematogenic pathways.^1^ In addition, TNM staging system included skull base perineural invasion as a T4 prognostic factor for non-melanoma skin cancer.^2^

The incidence of perineural invasion in basal cell carcinomas reported in the literature is usually under 1%, and it is generally associated with advanced cases.^3^, ^4^ In craniofacial resections, rates may reach 50%.^5^

The relationship between clinic pathological characteristics and perineural invasion is not fully understood. Most patients do not present with any specific symptoms, but paresthesia or pain may occur when there is gross nerve involvement.^6^ Skin tumors with perineural invasion were more frequently found in male gender, in the high-risk mask zone of the face, in larger and recurrent lesions, as well as in aggressive subtypes such as morpheaform and basosquamous.^7^

Major resections for basal cell carcinomas are often performed by a multidisciplinary team that includes a head and neck surgery specialist. In this setting, incidence of perineural invasion is expected to be high. This article reports the 18-year experience of head and neck surgery in a tertiary care referral center in São Paulo, Brazil, comparing advanced basal cell carcinomas with and without perineural invasion.

## Methods

The medical charts from patients submitted to surgery with head and neck surgery specialists in this institution from 1994 to 2012 were reviewed. Routine evaluation included CT scan prior to surgery. In all cases, general anesthesia was required; tumors were excised with appropriate margins, as confirmed by frozen section. Reconstruction was planned with local flaps, skin grafts, or muscular or microsurgical free flaps.

Data on gender, age, histological subtype of basal cell carcinoma, location, size, and perineural invasion were reviewed. Cases with tumors located in areas other than head and neck were excluded, as well as smaller lesions excised under local anesthesia.

Location was clinically defined as the center of the tumor, according to nine subsites: scalp, frontal, ear, periorbital, nose, malar, nasolabial fold, lip, and cervical. We also classified the risk assessment areas according to NCCN recommendations for skin neoplasm, which classifies high and moderate risk areas for head and neck topography.^2^ Size was measured after the resection by gross analysis of the specimen.

Basal cell carcinomas showing histological perineural invasion were compared with negatives cases. Statistical analysis was performed with Minitab 16 for Windows (Minitab Inc.; State College, PA, United States). The chi-squared test was used for qualitative data. Parametric variables were compared with Student *t*-test. Statistical significance was set to *p* < 0.05. The study protocol conformed to the ethical guidelines of the 1975 Declaration of Helsinki as reflected in the approval by the Institution Review under No. 0228/14.

## Results

Perineural invasion was positive in 82 cases out of a total of 354 basal cell carcinomas (23.1%). Demographic and clinicopathological data are shown in Table 1.

**Table 1:** Demographic and histopathological features of basal cell carcinomas with and without perineural invasion.

	Perineural invasion		
	Positive (*n* = 82)	Negative (*n* = 272)	*p*^a^
*Male gender* (*%*)	58.5	49.6	0.16
*Age* (*SD*)	65.4 (13.2)	65.0 (14.4)	0.8
*Size* (*cm*)	3.8 (2.2)	2.5 (1.8)	<0.01
*High-risk zone* (*%*)	88	88	1
*Ulceration* (*%*)	26.5	18.7	0.12

*Location* (*%*)			
Nose	35.7	36.9	0.85
Malar	21.1	20.2	0.72
Periorbital	16.1	13.1	0.6
Ear	7.6	8.3	0.85
Other	19.5	21.5	–

*Histological subtype* (*%*)			
Nodular	33.7	53.2	0.002
Morpheaform	48.2	26.4	<0.001
Basosquamous	6.0	4.4	0.54
Superficial	6.0	0	0.02
Micronodular	2.8	4.8	0.47
Others	7.3	7.2	–

*Mixed cases* (*%*)			
Yes	45.3	41.1	0.49
No	54.7	58.9	

a The chi-squared test was used for all variables, except age and size (Student's *t*-test).

## Discussion

Perineural invasion was defined by Batsakis as the presence of tumor cells in, around, and through the nerves.^8^ It was once considered as an angiolymphatic invasion to the nerve sheath,^9^ a hypothesis that was later rejected, since there is no lymphatic circulation there.^6^ Recently, neurotrophins and ligand–receptor complexes that effectively show a unique route of dissemination have been described. Some tumors are especially prone to perineural invasion and the reasons are not completely understood.^1^ In head and neck mucosal squamous cell carcinoma, perineural invasion is an independent prognostic factor, considered by some authors to be an indication for postoperative radiotherapy, even in the absence of lymph node metastasis.^10^ Nevertheless, such indication still remains a controversial issue in advanced skin tumors.^11^, ^12^

In basal cell carcinomas, perineural invasion, although rare, is an important prognostic factor,^13^ and it is rare.^3^, ^14^ Therefore, major resections that involve head and neck surgery specialists comprises a convenient selection of cases to study this particular issue.^15^ In the present study, a high incidence of perineural invasion was observed among basal cell carcinomas operated on in a tertiary care referral center, associated with morpheaform carcinoma and to larger tumors.

Men are more affected in most series reporting basal cell carcinomas in general,^16^, ^17^ and also in aggressive tumors with perineural invasion.^7^ Our results showed a non-significant higher preponderance of men.

Mean age in our series is similar to others, in which no difference was associated with more advanced tumors.^18^ Therefore, age is probably not associated with perineural invasion, as stated by this and other studies.^7^

On the other hand, larger tumor size was clearly associated with perineural invasion. Even in this selected series of major resections, average size almost doubles in tumors with perineural invasion. This is consistent with other series of basal cell carcinomas.^7^

Conversely, larger size was clearly associated with perineural invasion. Even in this selected series of major resections, the average size almost doubles in tumors with perineural invasion. This is consistent to other series of basal cell carcinomas.^7^

A controversial issue is the relationship between perineural invasion and location on the head and neck. At least in the present series, we found no difference either regarding risk zones or specific locations. Nasal and malar locations are the most frequent sites reported, but unlike other studies,^7^ we found no difference in frequency of perineural invasion in the different subsites. It is noteworthy, however, that almost 90% of the tumors were located in the high-risk mask zone of the face in both groups with positive and negative perineural invasion.

In the present study, the most important finding associated with perineural invasion was the histological subtype. Nodular basal cell cancer is the most common subtype among all basal cell carcinomas,^16^ and fortunately it is less frequently associated with perineural invasion. In the present series, superficial basal cell carcinoma was very rare, and no case demonstrated perineural invasion, as expected. However, morpheaform subtype, which accounts for only 6% of basal cell carcinomas in general, was the predominant subtype in almost half of the perineural positive cases in the present series.^19^ Although basosquamous subtype is recognized as aggressive,^20^ the incidence of perineural invasion was not particularly high among tumors of this subtype.

The limitation of this study is the selection of the cases, based on the referral of patients who needed a head and neck surgery specialist for a multidisciplinary surgical approach. However, the actual selection might be an indirect bias for aggressive tumors and major resections, helping to understand the main features related to perineural invasion. According to this series, perineural invasion is more commonly found in larger tumors and morpheaform subtype.

## Conclusion

Perineural invasion was clearly related to morpheaform subtype and to larger tumors. Other classically associated features, such as location in the high-risk mask zone of the face, male gender, and mixed histology, were not so strongly linked to perineural invasion.

## Conflicts of interest

The authors declare no conflicts of interest.
